# Perspectives on Deprescribing in long-term care: qualitative findings from nurses, aides, residents, and proxies

**DOI:** 10.1186/s12912-023-01179-y

**Published:** 2023-01-31

**Authors:** Milta O. Little, Emily J. Hecker, Cathleen S. Colon-Emeric, Laurie Herndon, Eleanor S. McConnell, Tingzhong Michelle Xue, Sarah D. Berry

**Affiliations:** 1 Duke University School of Medicine, Department of Medicine, Division of Geriatric Medicine, NC Durham, USA; 2 Durham VA Geriatric Research Education and Clinical Center, NC Durham, USA; 3 Hinda and Arthur Marcus Institute for Aging Research, Hebrew SeniorLife, Boston, MA USA; 4 Duke University School of Nursing, NC Durham, USA; 5 Beth Israel Deaconess Medical Center, Department of Medicine & Harvard Medical School, Boston, MA USA

**Keywords:** Deprescribing, Nursing home, Falls, Qualitative

## Abstract

**Background:**

Deprescribing initiatives in the long-term care (LTC) setting are often unsuccessful or not sustained. Prior research has considered how physicians and pharmacists feel about deprescribing, yet little is known about the perspectives of frontline nursing staff and residents. Our aim was to elicit perspectives from LTC nursing staff, patients, and proxies regarding their experiences and preferences for deprescribing in order to inform future deprescribing efforts in LTC.

**Methods:**

This study was a qualitative analysis of interviews with nurses, nurse aides, a nurse practitioner, residents, and proxies (family member and/or responsible party) from three LTC facilities. The research team used semi-structured interviews. Guides were designed to inform an injury prevention intervention. Interviews were recorded and transcribed. A qualitative framework analysis was used to summarize themes related to deprescribing. The full study team reviewed the summary to identify actionable, clinical implications.

**Results:**

Twenty-six interviews with 28 participants were completed, including 11 nurse aides, three residents, seven proxies, one nurse practitioner, and six nurses. Three themes emerged that were consistent across facilities: 1) build trust with team members, including residents and proxies; 2) identify motivating factors that lead to resident, proxy, nurse practitioner, and staff acceptance of deprescribing; 3) standardize supportive processes to encourage deprescribing. These themes suggest several actionable steps to improve deprescribing initiatives including: 1) tell stories about successful deprescribing, 2) provide deprescribing education to frontline staff, 3) align medication risk/benefit discussions with what matters most to the resident, 4) standardize deprescribing monitoring protocols, 5) standardize interprofessional team huddles and care plan meetings to include deprescribing conversations, and 6) strengthen non-pharmacologic treatment programs.

**Conclusions:**

By interviewing LTC stakeholders, we identified three important themes regarding successful deprescribing: Trust, Motivating Factors, and Supportive Processes. These themes may translate into actionable steps for clinicians and researchers to improve and sustain person-centered deprescribing initiatives.

**Trial registration:**

NCT04242186

**Supplementary Information:**

The online version contains supplementary material available at 10.1186/s12912-023-01179-y.

## Background

Polypharmacy has been associated with increased adverse outcomes, including falls and injuries [1–4]. Polypharmacy is even more prevalent in persons with dementia [5] and in the long-term care (LTC) setting, with the average resident being prescribed 8 medications [6]. Given the high risk of adverse drug reactions (ADRs) in this population and potential for reduced clinical benefit [7, 8], the importance of reducing the dose of medications or stopping medications (i.e., deprescribing) in LTC is great.

While successful deprescribing quality improvement initiatives in LTC have been created, they are often not sustained over time [9]. Previous studies have sought the input of physicians and pharmacists to identify barriers to deprescribing, including competing priorities for staff time and attention, lack of decision support tools, and ineffective educational strategies for frontline staff, such as didactics and passive dissemination [10–12]. While surveys have considered the perspective of nursing staff on deprescribing [13, 14], few studies have sought to identify facilitators of deprescribing from the perspective of LTC nurses, nurse practitioners, residents, and their designated healthcare proxies. Given that most direct care in LTC facilities is offered by nursing staff and most of the medical care is offered by nurse practitioners, it is critical to understand factors associated with deprescribing from the perspective of nurses, nurse aides, and nurse practitioners. Further, nursing staff and proxies are most likely to know what matters most to LTC residents, which is central to achieving patient-centered care while deprescribing. Therefore, our aim was to elicit perspectives from LTC nursing staff, including nurses, nurse aides (NAs), a nurse practitioner (NP), residents, and proxies (family member and/or responsible party) regarding their experiences and preferences for deprescribing to prevent injurious falls. These findings may be used to inform deprescribing initiatives in LTC.

## Methods

The research design utilized for this study was a qualitative analysis of semi-structured interviews. Study procedures, including the consent process, were performed in accordance with the Declaration of Helsinki and were reviewed and approved by the Institutional Review Boards of Hebrew Senior Life (Advarra) and Duke University. Informed consent was obtained from all participants and/or their legal representatives. Study reporting is consistent with the Association of American Medical Colleges Standards for Reporting Qualitative Research (SRQR).

Two female team members (EH, LH) conducted onsite interviews with LTC staff (nurses, nurse aides, nurse practitioner), residents, and proxies in three facilities. Interviewers had a background in nursing and had no previous relationship with the staff. Because prior qualitative studies focused on the perspectives of physicians and pharmacists, we omitted these disciplines in our sample. Facility size varied from 60 to over 100 beds and all facilities were non-profit in urban or suburban locations. Two facilities were academically affiliated, and two accepted Medicaid. Details of recruitment and facility characteristics have been previously published [15, 16]. In brief, residents at high risk for fractures due to a recent fall were identified and the study team was provided a list of their proxies and full-time staff. A purposive sampling strategy was used to ensure that at least 25% of participants were from underrepresented racial and ethnic groups, and approximately one-third of the sample represented residents/proxies, NAs, and nursing staff, respectively, as these were the primary informants of interest to this analysis.

Interview guides were designed to inform components of an injury prevention intervention, including deprescribing and osteoporosis treatment (Additional file 1 Appendix 1). Results of the analyses of osteoporosis treatment and injury prevention have already been published [15, 16]. Interviews were audio recorded and transcribed. We employed a qualitative framework methodology to translate the transcribed interviews into core themes that described the participant experiences and preferences with deprescribing [17]. This framework methodology has been used by interprofessional research teams to generate hypotheses to improve clinical practice [18, 19]. Thus, it was a well-suited methodology to achieve our goal of informing an injury prevention intervention. Participant enrollment continued until theme saturation was reached, which was identified by grouping coded memos by specific domains (structure, process, outcome, and touch point) and subsequently identifying themes, ensuring themes were inclusive of all interviews with agreement between interviewees. No differences were discovered between groups or between geographic location. We made sure that themes were inclusive of all interviews, and that we found agreement between interviewees regarding the identified themes. Each theme was reviewed in this manner by at least two members of the team, and then findings were reviewed by the entire team.

**Fig. 1:**
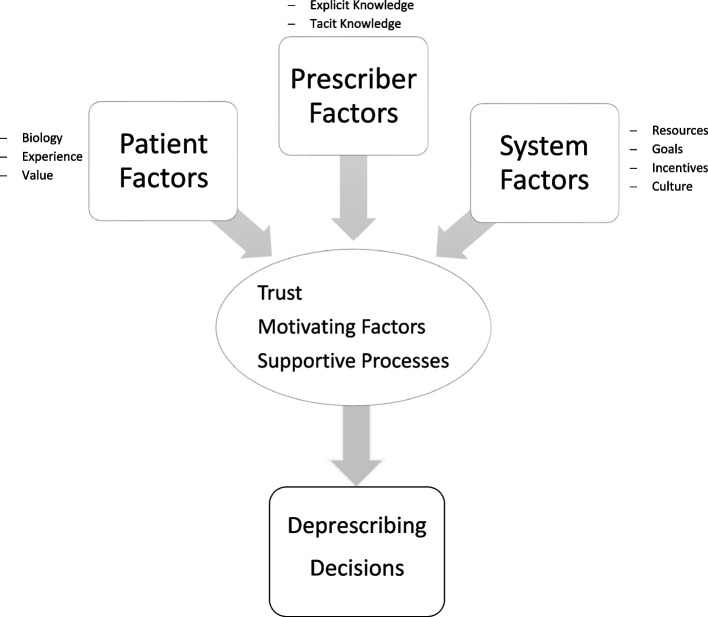
Key themes (circle) that lead to deprescribing decisions emerged after applying Desprescribing Conceptual Framework [17] Domains (top boxes) to staff, resident and proxy inteviews in three long-term care facilities

First, team members read the interviews. Second, we categorized participant responses using themes in NVivo 12™. Team members met regularly to refine codes; at least two team members coded each transcript. Third, we used the **Deprescribing Conceptual Framework** to map the themes to a domain [20] (Fig. 1). The Deprescribing Conceptual Framework was developed to organize deprescribing research agendas and can be expected to translate well into the LTC setting given the interdependency of patient, prescriber, and system factors for care decisions and the patient-centered movement in long-term care. The framework acknowledges that the decision to deprescribe is influenced by patient factors, prescriber factors, and system factors. Further, the framework considers nine shared and non-shared decision-making domains that influence deprescribing. Each quote was mapped to a corresponding framework domain. Summary statements were created to capture the messages the quotes contained. A team member [MOL] reviewed results to ensure code assignment and summary statements were complete. Next, overarching themes were identified. The full study team reviewed the themes to ensure they were consistent with quotations and summary statements. Finally, the team developed a list of actionable clinical implications related to each theme. Actionable items were developed based on the team’s interpretation of the staff interviews while using their clinical and research expertise These actionable implications were compared with domain quotes to ensure that they captured the voice of the nursing home workers and patients. All members of the team were involved in developing the themes and actionable implications to ensure the trustworthiness of our study results.

## Results

We enrolled participants from three LTC facilities in two states (MA, NC). 26 interviews with 28 participants were completed, including 11 NAs, three residents, seven proxies, one nurse practitioner, and six nurses. Two interviews involved resident-proxy dyads. Table 1 lists participant characteristics.

**Table 1:** Characteristics of participants interviewed regarding deprescribing preferences in three long-term care facilities

Characteristic	***N*** = 28
Median age (IQR)	
Residents (*n* = 3)	81 [79,88]
All others (*n* = 25)	58 [36,66]
Female (%)	23 (82)
Race/Ethnicity (%)	
White	16 (57)
Black	9 (32)
Hispanic	3 (11)
Role (%)	
Resident	3 (11)
Proxy	7 (25)
NA/Rehab Aide	11 (39)
Nurse	6 (21)
Nurse practitioner	1 (4)

Three themes related to deprescribing emerged (Fig. 1): 1) develop trust with team members, including residents and proxies; 2) identify motivating factors leading to deprescribing acceptance; 3) standardize supportive processes that encourage deprescribing. These themes are summarized in Table 2, with actionable steps**.**

**Table 2:** Key themes and actionable steps for deprescribing in long-term care

Themes	Examples	Actionable Steps
Trust among team members	Recommendation from trusted physicians are critical for residents and caregivers	1. Share deprescribing data with frontline staff 2. Tell deprescribing stories to staff, residents, and caregivers
	Previous experiences (deprescribing without adverse effects or failing to deprescribe with adverse effects) are powerful	
	Frontline staff and proxies inform prescribers of condition changes	
	Proxy and staff express concerns regarding medications to team members	
Motivating factors leading to deprescribing acceptance	Explicit and tacit understanding of risk of ADRs/side effects in the LTC population	3. Provide geriatric-pharmacology education to frontline LTC staff 4. Align medication risk/benefit discussions with what matters most to the resident
	ADR may be the cause of a condition change or fall	
	The desire to maintain independence	
Processes that support deprescribing	Care plan meeting is an opportunity to discuss medications and medication-related concerns	5. Standardize deprescribing monitoring protocols 6. Standardize interprofessional team huddles and care plan meetings to include deprescribing conversations 7. Explicitly build deprescribing opportunities into the existing workflow at points of transitions and during falls assessments using scripts or templates 8. Strengthen non-pharmacologic treatment programs
	Nursing and proxy reports to nurse practitioners and physicians result in deprescribing	
	Availability of non-pharmacologic alternatives can support deprescribing	
	Falls must be reported to physicians and nurse practitioners, and this may trigger an interprofessional medication review	

### Trust with team members

The Deprescribing Conceptual Framework [20] highlights the important relationship between the patient and physician or nurse practitioner in making deprescribing decisions. Our study shows that trust, which is built through mutual respect and active listening, between healthcare workers AND between the resident or proxy and the healthcare team is a critical component of this relationship. Stakeholders identified multiple examples of factors that build trust with team members.

First, stakeholders noted that residents and caregivers are often willing to accept deprescribing when it is recommended by a trusted physician, backed by experience or data.

*“If the doctor said anything to her, she’d just say okay.” (Proxy) “And I think [the doctor’s] opinion counts. That’s how I feel about it.” (Resident).*

Second, previous experiences with deprescribing may influence the willingness of nurse practitioners and patients to deprescribe in the future. Stakeholders described successful instances of deprescribing:

*“Over time, she was taken off of [all her medications]. And she’s now taking only Tylenol … and doing fine.” (Nurse Practitioner).*

*“There was a resident that … was having some falls, and at one point really wasn’t ambulating that much …*. *And they did reduce the patient’s meds, and she is ambulating pretty much on her own” (NA).*

Third, stakeholders in all three facilities observed that it is the frontline nursing staff and proxies who inform medical prescribers of condition changes due to adverse drug reactions, who trust that they will speak up.

*“we are the eyes of - eyes for … the doctors. So, we have to monitor every changes on the … residents.” (Nurse).*

*“You have to have a good eye to know your … residents. And I knew something wasn’t right. Hey, that’s not his normal. Let’s see what’s going on.” (NA).*

*“I noticed her increasing sleepiness, and I was afraid it was the pain medicine, and said, can we just gently pull back on it. And we did.” (Proxy).*

Last, proxies related stories of expressing strong concerns regarding medications and a desire to talk with the nursing staff as well as the physician and nurse practitioners.

*“She was on Ativan … that was a big problem with me and the nurses …. I went to her doctor and even said, ‘You got to get her off.’ …*.” *(Proxy).*

*“I would talk to the nurse and then both doctors. And then the nurse practitioner. I’d talk with all of them.” (Proxy)*

### Motivating factors

Each member of the team may identify motivating factors for deprescribing acceptance, based on past experiences, personal values, and knowledge base, both tacit and explicit. In addition, system factors that promote deprescribing, such as available resources, aligned goals, and a team-based care culture, may lead to greater acceptance of deprescribing. Our interviewees identified several motivating factors for deprescribing acceptance. For example, a practitioner noted that the explicit knowledge that lower doses of medications may be required due to the increased risk of ADRs in older adults may lead to closer patient monitoring by staff and subsequent deprescribing of risky medications by prescribers.

*“Well, we have a lot of … patients in 80s and 90s, and we are in a facility directed by a geriatrician. So, we’re very conscious of not wanting to push the blood pressure too low. And so … when we have a fall, especially, we’re evaluating blood pressures, sitting, standing.” (Nurse Practitioner).*

Frontline staff identified that the presence of a change in condition may be related to a medication ADR. Close symptom monitoring and awareness that a change in condition can be the result of an ADR helps to transform tacit knowledge to explicit knowledge for frontline staff, and it may also be an important driver of deprescribing.

*“A resident might be active all the time. Then suddenly, you walk in the room, and they might be sleeping. Or dozing off, … we have to mention it to the nurse. That ‘This person is kind of inactive,’ and the nurse might be like, ‘There is a change in her medication, so that is why.’” (NA).*

*“On the floor, they are just following orders, and they oftentimes don’t even know why it’s being ordered. They will just call and say, “We are upping your mom’s pain medication”. It’s stated as a statement. And that’s not the way I want to hear it. “We are upping your mom’s pain medication because …” , and here are the pros and cons …. Context matters.” (proxy).*

Aligning deprescribing conversations with what matters most to the patient (i.e., goal-concordant care), may increase the success of deprescribing initiatives. Past experiences with deprescribing that were successful in meeting care goals were described as motivating factors for accepting future deprescribing.

*“My mom was desperate to be independent - and so if you threatened her with, ‘If you don’t do this, you will have to go into the nursing home … ’”* (while discussing effective ways to encourage her mother to reduce benzos) *(Proxy).*

“*… she began to … take on this glassy-eyed look and sort of a flat expression. And … she had a tremor with her hand … so, I took her to a neurologist who did all the little pre-tests. And then she said, “Let me take a look at her med list,” and … she’d said, “Here’s the problem right here,” and it was a drug interaction. Got rid of one of them, and it all went away. So, after that experience, you know, … I was kind of the one that wanted them to give her less medication than they really wanted to give her, to be honest.”* (Proxy)

### Supportive processes

Deprescribing may take place more readily when the care environment uses supportive processes to act on interprofessional staff observations, has adequate resources to support non-pharmacological treatment and systems in place to encourage routine deprescribing, such as nurse practitioner review after a fall. Interviewees identified several opportunities or missed opportunities to improve processes for initiating and sustaining deprescribing in long-term care. First, the care plan meeting is a key LTC process that provides an opportunity to discuss medication concerns and identify deprescribing opportunities.

*“That’s why the care plan is so important. A care plan of a person, knowing the person’s care plan, helps you understand what needs to happen next, you know.” (NA).*

*“In the family care meetings, the people who are in the meeting are usually not the people who work with her every day …. It’s not an aide or a nurse that works with her every day … so when I do have questions, a lot of times they can’t answer them.” (proxy).*

Second, it was noted that physicians will deprescribe medications based on resident reports given by nurses. Explicit education to nursing staff and proxies may facilitate deprescribing events.

*“So, if we see that a medication is causing an adverse side effect, we do bring it to the doctor’s attention. And then, they’ll add parameters on it - or they’ll say let’s taper or let’s DC [discontinue] it and see how it goes.” (Nurse)“I feel like the nurse should talk to both the doctor and the nurse practitioner, because the nurse is with [the resident] more than the doctor and the NP, so she should have a big role in what they say.” - proxy.*

Third, multiple stakeholders identified that supportive processes, including the availability of non-pharmacologic alternatives, can lead to sustained deprescribing.

*“Well, if you have to take something away, you have to add something, I feel. And you know, activities would be the thing!” (Nurse).*

*“… my concern would be if you didn’t have to give a person the drug, and other ways that we could meet their needs, maybe through life enrichment, or some other capacity - would that be a choice, rather than the drug?” (NA).*

Finally, interviewees noted that falls may trigger an interprofessional medication review, increasing opportunities for deprescribing and deprescribing education.

*“There is a committee that reviews falls after they happen … They ask the medical staff at times … , to be involved with evaluating is there medication that could be affecting the falls risk.” (Nurse Practitioner).*

## Discussion

Deprescribing initiatives have been shown to improve outcomes for LTC residents [4, 21]. Most recently, a meta-analysis of deprescribing interventions in nursing homes found deprescribing interventions reduced mortality by 26% and the number of fallers by 24% [21]. These studies primarily engage physicians and pharmacists in the deprescribing process, and our study suggests that engagement of nursing staff (including aides), residents, and proxies, who know what matters most to LTC residents, is central to achieving *sustained* patient-centered deprescribing in this setting. A recent publication describing factors related to sustainability of quality improvement projects in care homes reinforces the importance of engaging and supporting frontline staff [22]. Through interviews with front-line nurses, nurse aides, a nurse practitioner, residents, and proxies, we identified three themes related to their experiences with and preferences for deprescribing: 1) develop trust with team members; 2) identify motivating factors leading to the acceptance of deprescribing; 3) standardize individual and system supportive processes that encourage deprescribing. These themes highlight that successful deprescribing in LTC should not only involve pharmacists and prescribers, but should involve all members of the team, including front-line nursing and NAs. Further, these themes suggest several important practices, including storytelling and standardizing deprescribing processes, that should facilitate successful deprescribing programs.

### Trust

Successful and sustained quality improvement in LTC is predicated on the need to develop trusted therapeutic relationships among all members of the team, including nurses, residents and caregivers. Trust begins with mutual respect and active listening. Previous positive experiences with deprescribing likely influence the willingness of physicians, nurse practitioners, and residents to deprescribe in the future and these experiences can be used to build trust among the team and improve buy-in for deprescribing. Our findings suggest that residents and caregivers are more likely to agree to deprescribing if they trust the physician or nurse practitioner and feel that their opinion has been heard. An Australian study that included 35 interviews from nursing home physicians and nurses supports this finding as they concluded the quality of interprofessional collaboration and communication with families is a major factor in successful care delivery [23].

### Motivating factors

Physicians and nurse practitioners act daily on tacit knowledge, and supplementing with explicit knowledge can help guide deprescribing efforts. The LTC stakeholders in this study identified key themes leading to acceptance of deprescribing efforts that could be incorporated into formal interprofessional educational opportunities. Examples of explicit knowledge that could be gained through interprofessional educational exchanges includes ensuring that frontline nursing staff, residents, and proxies understand the relationship between deprescribing and falls prevention, the potential ADRs in high-risk medication classes, and the increased risk of ADRs in older adults.

### Processes

Standardizing individual and system processes and triggers for deprescribing help to provide high-value opportunities for engaging in shared decision making. Our findings suggest that system processes can be built to trigger a medication review at points of transition and after an injurious fall. Despite the connection between polypharmacy and falls, in community dwellers with a fragility fracture, medications associated with falls are seldom deprescribed in the months following the injury [24]. In the LTC setting, it is mandated that nursing staff report every fall to a nurse practitioner or physician; however, it is unclear whether this reporting practice facilitates a medication review or deprescribing. Facility leaders should explicitly consider deprescribing processes through “if-then” planning interventions [25] during transitions of care huddles, care plan meetings, and post-fall evaluations in an effort to minimize future falls. For example, *if* a resident experiences a non-injurious fall, *then* the nurse will review the medication list using a checklist and report any high-risk medications to the physician or nurse practitioner.

In addition, patient-centered care requires that we elicit care goals prior to making treatment decisions. Following a standard patient-centered process to ensure goal-concordant care, which starts with understanding what matters most to the resident and proxies, was also an important theme in this analysis, which is consistent with deprescribing models described in the literature that emphasize the alignment of patient goals with medication management goals [26–30]. In LTC, it is often the proxy, not the resident, who establishes these goals. Care plan meetings often involve proxy and resident participation and if not used as an opportunity for person-centered interprofessional team communication, this represents a missed opportunity in a standard LTC process to include residents’ goals and personalized risk/benefit discussion to align medication prescribing/deprescribing with their preferences.

Additionally, our findings suggest that deprescribing episodes are more likely to be successful and sustained if there are concurrent processes to initiate non-pharmacologic alternatives to medications, such as comprehensive activities programming. Having these readily available to residents may help to engage all members of the team in identifying deprescribing opportunities.

### Limitations and strengths

Some limitations exist in this study. First, qualitative data provides rich information to elucidate important themes, but allows only for hypothesis generation. It remains to be studied whether the recommendations stemming from this analysis will result in meaningful deprescribing process models that will lead to reduction of injurious falls. Second, we did not include physicians or pharmacists, which are key stakeholders in deprescribing, in the interviews. Much of the existing literature on deprescribing in LTC concerns physicians and pharmacists and so we sought to instead capture the nursing, patient, and proxy perspective. Third, the Deprescribing Conceptual Framework was developed to organize deprescribing research agendas, but to our knowledge, it has not been used in the long-term care setting. Nevertheless, the framework domains can be expected to translate well into this setting given the interdependency of patient, prescriber, and system factors for care decisions and the patient-centered movement in long-term care. Strengths of the study include the use of a multi-step qualitative analysis process using a validated deprescribing framework and the inclusion of a wide spectrum of views, representing multiple members of the healthcare team.

## Conclusions

In conclusion, by interviewing nursing staff, residents, and proxies, we identified three important themes regarding successful deprescribing for LTC residents: Trust, Motivating Factors, and Processes. These themes may translate into the following eight actionable steps for clinicians and researchers to improve and sustain deprescribing initiatives: 1. Share deprescribing data with frontline nursing staff, 2. Tell stories about successful deprescribing to staff, residents, and caregivers, 3. Provide deprescribing education to frontline LTC staff, 4. Align medication risk/benefit discussions with what matters most to the resident, 5. Standardize deprescribing monitoring protocols, 6. Standardize interprofessional team huddles and care plan meetings to include deprescribing conversations, 7. Explicitly build deprescribing opportunities into the existing workflow at points of transitions and during falls assessments using scripts or templates, and 8. Strengthen non-pharmacologic treatment programs. These implications provide opportunities for researchers and policymakers to improve person-centered deprescribing processes for long-term care residents.

## Supplementary Information

**Additional file 1: Appendix 1.** Interview Guide – NH PRIDE Fall Prevention Experience.

## Data Availability

This study includes the collection of qualitative data from a small number of facilities; therefore, we do not have plans to create a publicly available dataset. Further, our original consent of participants did not include permission to make the qualitative data publicly available. If another investigator requests the data, they should reach out to the senior author (SB) who will work with the investigator to make the data available, to the extent allowable by our IRB and institutional data sharing policies.

## Abbreviations

LTC: long term care
ADR: adverse drug reaction
NAs: nurse aides
