# Supplementary material for: Perspectives on Deprescribing in long-term care: qualitative findings from nurses, aides, residents, and proxies
Source: BMC Nurs. 2023 Jan 31;22:27. doi: 10.1186/s12912-023-01179-y (PMC9890706; doi:10.1186/s12912-023-01179-y)
Supplement: Supplementary file 1 — Additional file 1: Appendix 1. Interview Guide – NH PRIDE Fall Prevention Experience. [file 12912_2023_1179_MOESM1_ESM.docx]

**Grand Tour Question**: Please tell me about a time that you remember when [you/your family member/one of your residents] had a fall.

Probe:

1. How did the fall affect [you/your family member/your resident]?
2. We are interested in learning about how the things we do to prevent falls and injuries in nursing homes impact patients, families, and staff. Please tell us what was done for [you/your family member/your resident] to try to prevent falls or injuries either before or after that fall.

Probes:

1. What surprised you about the things that were done to prevent falls or injuries?
2. What was most troublesome?
3. What were you most pleased about?
4. What do you wish had been done differently?
5. What did you hope would happen as a result of the plans that were put into place?
6. One of the things we often recommend for people at risk for falls is to reduce or stop medications that make them sleepy or make their blood pressure drop too low. Please tell us about a time when a doctor or nurse practitioner wanted to reduce or stop one of [your/your family member’s/your resident’s] medications. [If the interviewee cannot recall a specific example, ask them to imagine that a provider wanted to reduce/stop a medication they had been taking for depression, anxiety, sleep or blood pressure]

Probes:

1. How [did/would] you feel about the suggestion?
2. (For residents/proxies): Whom would you want to discuss these suggestions with? (e.g. pharmacist, nurse practitioner, unit nurse)
3. What questions [did/would] you have?
4. What [did/would] you worry might happen? [If recounting a real story] What did happen?
5. What would have reassured you of your concerns?
6. If you found yourself in this situation, what would it take to get you to agree to stopping the medicine?
7. Another thing we sometimes recommend to prevent broken bones after a fall is treating osteoporosis, or weak bones, with medications such as Fosamax (alendronate). Has a medical provider talked to [you/your family member/one of your patients] about that?

Probes:

1. How [did/would] you feel about the suggestion?
2. What questions [did/would] you have? What would help you make a decision?
3. What [did/would] you worry might happen? [If recounting a real story] What did happen?
4. What would make you hesitant to take the medication?
5. Would you make a different decision if they told you it would decrease the chance of having a hip fracture by 1/3 to 1/2?
6. Preventing falls and injuries takes a lot of coordination between the resident, family, nursing staff, medical team, and pharmacist. Please tell us how communication between you and these other people works in your nursing home.

Probes:

1. (For residents/proxies): Whom do you prefer to communicate treatment recommendations? (e.g. nurse practitioner, physician, unit nurse, specialty nurse)
2. Have you had the experience of communicating with a nurse specialist who makes recommendations to the team? Examples might include a wound care nurse, an infection control nurse, or a palliative care nurse. How did you feel about this interaction?
3. What have you experienced that makes communication better or easier?
4. What makes it more difficult?
5. What additional ideas do you have to improve communication between yourself and all these different people?
6. (For staff only): What experiences have you had using telehealth or case-based discussions to improve the quality of care for your residents? If not, how would you feel about using telehealth or case-based discussions.
